# Supplementary material for: Improvement of violence management among nurses in Iran: The best practice implementation project in a health promoting hospital
Source: PLoS One. 2023 Nov 27;18(11):e0284758. doi: 10.1371/journal.pone.0284758 (PMC10681186; doi:10.1371/journal.pone.0284758)
Supplement: Attachment — Submitted filename: Response To reviewers.docx [file pone.0284758.s001.docx]

**Reviewer #1: This study aims to identify and provide strategies against workplace violence among nurses in a single small-scale hospital in Iran. The topic is important in practice. Its strength is including pre- and post- intervention evaluations, and the policy implementation was performed in real-life workplace. I have a few minor comments and I hope the authors will find them useful for improving this manuscript.**

1. We notice that your manuscript file was uploaded on May 31 2022. Please can you upload the latest version of your revised manuscript as the main article file, ensuring that does not contain any tracked changes or highlighting.

**Both of highlighting manuscript and main manuscript were attached .**

2. Please include a separate legend for Figure 1 in your manuscript.

**It was revised.**

3. Please ensure that you refer to Table 3 in your text as, if accepted, production will need this reference to link the reader to the Table

**It was referred in text.**
